# Effect of ranolazine on Tp‐e interval, Tp‐e/QTc, and P‐wave dispersion in patients with stable coronary artery disease

**DOI:** 10.1002/joa3.12549

**Published:** 2021-05-17

**Authors:** Murat Akcay, Metin Coksevim, Mustafa Yenercag

**Affiliations:** ^1^ Department of Cardiology Faculty of Medicine Ondokuz Mayis University Samsun Turkey; ^2^ Department of Cardiology Faculty of Medicine Ordu University Ordu Turkey

**Keywords:** coronary artery disease, index of cardiac electrophysiological balance (iCEB), P‐wave dispersion, ranolazine, Tp‐e interval

## Abstract

**Introduction:**

Ranolazine is an antianginal drug and also exhibits antiarrhythmic effect by affecting action potential time, refractory period, and repolarization reserve. We evaluated the effect of ranolazine therapy on myocardial repolarization parameters (Tp‐e, QT, QTc intervals, Tp‐e/QT, and Tp‐e/QTc ratios), index of cardiac electrophysiological balance (iCEB) (QT/QRS, QTc/QRS) and P‐wave dispersion (PWD) in patients with stable coronary artery disease (CAD).

**Methods:**

This study included 175 patients, aged between 35 and 90 years who were followed with stable CAD for at least 3 months. Ninety patients had been receiving ranolazine for at least 1 month, and 85 patients had never received ranolazine. All patients' basic demographic data, risk factors, medications, and echocardiographic parameters recorded. Myocardial repolarization parameters, P‐wave times, and PWD were analyzed from 12 lead electrodes.

**Results:**

There was no variation between the groups in terms of basic demographic parameters and CAD risk factors. Tp‐e interval (87.3 ± 14.4 vs. 90.8 ± 12.4 msn, *P* < .001), Tp‐e/QT (0.22 ± 0.04 vs. 0.23 ± 0.03; *P* = .03), Tp‐e/QTc (0.21 ± 0.04 vs. 0.22 ± 0.04 *P* = .001), and PWD (39.2 ± 13.7 vs. 43.5 ± 12.9 *P* = .028) were significantly lower in the ranolazine group. But iCEB was similar in both groups. In multivariate analysis after adjusted confounding factors such as age and BMI, Tp‐e/QTc ratio, QTc, P_max_, and PWD were found significantly in ranolazine group again.

**Conclusion:**

Tp‐e/QTc ratio, QTc, P_max_, and PWD were significantly lower in stable CAD patients under ranolazine therapy. In stable CAD patients, the prognostic significance of ranolazine for arrhythmic events requires further evaluation of these parameters through long‐term follow‐up and large‐scale prospective studies.

## Graphical abstract

Ranolazine leads to asymptomatic cardiac electrocardiographic changes in stable coronary artery disease patients. Myocardial repolarization parameters (Tp‐e interval, QTc interval, Tp‐e/QT, Tp‐e/QTc ratios) and P‐wave dispersion (PWD) were significantly lower in stable CAD patients receiving ranolazine therapy.

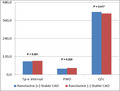

## 1 INTRODUCTION

Ranolazine is a drug produced from piperazine, which provides anti‐ischemic efficacy without a clinically important effect on heartbeat or blood pressure.^1^ Ranolazine inhibits intracellular sodium (Na) increase by inhibiting the late Na inflow channels and accordingly because of Na–Ca ion exchange, prevents indirect intracellular calcium (Ca) accumulation. Ranolazine reduces ventricular diastolic tension and oxygen consumption with this reduction in cellular Ca accumulation and reduces symptoms in patients with stable angina pectoris. In addition, a decrease in the incidence of arrhythmias is expected because of this mechanism of action.^1^, ^2^ Its effects are independent of hemodynamic parameters, unlike beta blockers, calcium channel blockers and nitrates.^1^ Ranolazine may cause dose‐dependent increase in corrected QT interval (QTc), decrease in T‐wave amplitude and rarely T‐wave notching. Patients received ranolazine were observed reduction in attacks of supraventricular tachycardia, new onset atrial fibrillation (AF) and ventricular tachycardia (VT).^3^, ^4^ In selected patients, ranolazine may be utilized for anti‐arrhythmic effect in addition to antianginal effect, but it is not indicated for only anti‐arrhythmic effect^2^, ^3^

The interval among the peak to end of the T wave (Tp‐e) is a measurement of trans‐myocardial dispersion of repolarization and may be related with malign rhythm disorders and ventricular arrhythmias^5^, ^6^, ^7^ Index of cardiac electrophysiological balance (iCEB) was described as QT time divided by QRS time (QT/QRS) from the electrocardiogram. It is a new and noninvasive feature, which may anticipate malign ventricular arrhythmias.^8^ Again, P‐wave dispersion (PWD) defined as the prolongation of inter‐atrial and intra‐atrial transport duration of sinus rhythm is well defined electrophysiological feature as a precursor of AF.^5^, ^6^, ^7^

In our study, we aimed to detect the abnormal findings and effect of ranolazine on repolarization parameters (Tp‐e interval, Tp‐e/QT, Tp‐e/QTc, QT, QTc), iCEB, and PWD in patients with stable coronary artery disease (CAD).

## 2 METHODS

### 2.1 Study population and protocol

This prospective study included a total of 175 patients, aged between 35 and 90 years, who were followed with stable CAD for at least 3 months. The patients were split into two groups as 90 patients (Group 1) receiving ranolazine and 85 patients (Group 2) not receiving ranolazine. Patients in group 1 had been on ranolazine for at least one month, while patients in Group 2 had never received ranolazine before.

As exclusion criteria, history of hypertrophic or restrictive cardiomyopathy, acute coronary syndrome patients, left ventricular ejection fraction < 40%, severe obesity (BMI ≥ 40 kg/m^2^), severe heart valve disease (severe aortic stenosis and insufficiency, severe mitral stenosis, and insufficiency), poor echocardiographic image quality, constrictive pericarditis, severe pericardial effusion, AF, QRS duration ≥ 120 msn, pregnancy, severe liver and renal failure, severe anemia, severe metabolic, and electrolyte abnormalities were accepted. All patients were informed about study and taken written permission. The protocol was accepted by the ethics committee of the Faculty of Medicine, Ondokuz Mayis University (No: 2020/119) and adhered to the Declaration of Helsinki (2013version).

Basic demographic data such as age, gender, height, weights, systolic and diastolic blood pressure (SBP and DBP), diabetes mellitus (DM) type II, hypertension (HT), hyperlipidemia (HL), smoking, medications, and history of myocardial infarction were recorded. SBP and DBP of all patients were measured from the brachial artery with a sphygmomanometer after resting for at least 10 minutes. Body mass index (BMI) was measured by dividing weight in kilograms by the square of the height in meters (kg/m^2^). Biochemical blood evaluation was performed after fasting for 12 hours.

### 2.2 ECG analysis

Electrocardiography (ECG) papers were printed in a quiet room after 5‐minute rest, with 20 mm/mV of amplitude and 50 mm/s of rate from an ECG machine (Cardiovit AT‐102 ECG, Schiller, Switzerland). All ECG papers were analyzed and transferred to computer. Adobe Photoshop program was used for 200% amplification the parameters. ECG parameters were analyzed by blinded two different cardiologists. When differences were detected between the measurement, the analysis was repeated by two cardiologists and mean values were taken.

### 2.3 Measurement of Tp‐e, QT, and QTc intervals

Tp‐e, QT and QTc intervals were analyzed from superficial ECG papers. All 12 leads were analyzed but usually assessed in leads DII and V5. The unipolar chest leads usually reflect trans‐myocardial distribution of repolarization, but bipolar extremity leads reflect the global distribution of repolarization, covering apical‐basal and interventricular dispersion ^5^, ^6^, ^7^, ^9^.

Tp‐e interval described as time between the highest amplitude of the T wave to end of the T wave according to the isoelectric line. The QT interval was calculated as distance between the starting of the QRS to end of the T wave according to isoelectric line. The R‐R distance was measured and used to compute the heart rate and to correct QT (QTc) distance with Bazett's formula (QT interval/√[RR interval]). Again, iCEB was obtained from QT time divided by QRS time (QT/QRS). All parameters were repeated three times and the mean values were recorded for each ECG parameter.

### 2.4 PWD measurement

The onset and end of P wave were determined as the point first and last deflection tip of the P wave intersected the isoelectric line. The maximum P wave (P_max_) and minimum P wave (P_min_) time were measured from all ECG derivations. PWD was calculated as the difference among the P_max_ and the P_min_ time.

### 2.5 Echocardiography

The echocardiographic examination was performed in the left lateral decubitus position from standard acoustics views with Vivid 7 device (GE Medical System, Horten, Norway; 3.5‐MHz phased array transducer). The left atrial diameter, left ventricle end‐diastolic and end‐systolic dimensions, interventricular septum, and posterior wall thickness were measured in parasternal long‐axis view. Left ventricular mass was measured using the Devereux's formula and indexed to body surface area (BSA).^10^ LA volume was measured using the biplane area‐length technique in the apical 4‐chamber view and indexed to BSA. Ejection fraction was measured by modified Simpson method. M‐mode, two dimensional (2‐D), Doppler, and colored Doppler echocardiographic parameters of all patients were obtained and evaluated according to American Society of Echocardiography standards.

### 2.6 Statistical analysis

The study parameters were transferred to a computer and assessed via IBM SPSS 20 (SPSS Inc, Chicago, IL, USA). Descriptive statistics were described as mean ± standard deviation, median (minimum‐maximum), numbers, and percentages. Chi‐square test and Fisher exact test were used to compare categorical values. The suitability of the values to normal dispersion was evaluated by visual (histogram and probability graphs) and analytical techniques (Kolmogorov–Smirnov test). For variables that were found unsuitable for normal dispersion, Mann–Whitney U test for statistically significant distinction among two independent groups was used, whereas Student's t test was used for normally distributed parameters. In multivariate analysis, independent effects of possible factors (age, BMI, and heart rate) on the use of ranolazine were examined using logistic regression analysis. Hosmer–Lemeshow test was used for model adaptation. The parameters were determined using the enter method. Statistical significance level was accepted as *P* value <.05.

### 2.7 Reproducibility

Intraobserver and interobserver variability for Tp‐e, QT, QTc, P_max_, and P_min_ values were assessed by repeating the measurements of 60 randomly selected individuals with 30 subjects from the ranolazine (+) group and 30 subjects from the ranolazine (−) group. These parameters were re‐evaluated by the same physician at least 1 month later for intraobserver variability and by another one blinded to the data of the subjects for interobserver variability. Reproducibility analysis was measured with intraclass correlation coefficient (ICC). Intraobserver and interobserver variability was lower than 5% and nonsignificant (*P* > .05) for all ECG parameters.

## 3 RESULTS

Demographic data for ranolazine and control groups are summarized in Table 1. There was no statistically important variation between the groups in terms of gender, SBP and DBP values, HL, DM type II, cigarette, previous MI, and stable CAD status (*P* > .05).

**TABLE 1:** Basic demographic characteristics and risk factors of patients

Variable	Ranolazine (+) CAD group	Ranolazine (−) CAD group	*P* value
	(n = 90)	(n = 85)	
Age (year)	67.1 ± 9.2	64.8 ± 8.7	**0.030**
Gender			
Men, n (%)	60 (66.7)	59 (69.4)	0.697
Women, n (%)	30 (33.3)	26 (30.6)	
BMI (kg/m^2^)	27.4 ± 4.1	29.1 ± 4.3	**0.011**
Systolic BP (mmHg)	129.9 ± 10.3	130.5 ± 12.9	0.542
Diastolic BP (mmHg)	80.3 ± 9.7	79.8 ± 8.7	0.625
Heart rate (bpm/min)	71.9 ± 10.8	68.0 ± 9.3	**0.008**
Cigarette, n (%)	24 (26.7)	17 (20.0)	0.298
Hypertension, n (%)	76 (84.4)	57 (67.1)	**0.007**
Hyperlipidaemia, n (%)	38 (42.2)	31 (36.5)	0.436
Diabetes mellitus type II, n (%)	35 (38.9)	27 (31.8)	0.325
Stable CAD, n (%)			
Stent	49 (54.4)	53 (62.4)	0.407
Bypass	23 (25.6)	21 (24.7)	
Stent + bypass	18 (20.0)	11 (12.9)	
Prior MI history, n (%)	51 (56.7)	49 (57.6)	0.896

Note

Continuous variables are presented as “mean ± SD and median (minimum‐maximum)” and categorical variables as “number (percentage).”

Abbreviations: BMI, body mass index;BP, blood pressure; CAD, coronary artery disease; DM, diabetes mellitus; MI, myocardial infarction.

Bold values indicate statistical significance.

The age and heart rate of patients receiving ranolazine were significantly higher than those without ranolazine, whereas BMI was significantly lower (*P* = .030; *P* = .008; *P* = .011, respectively). In addition, HT was importantly higher in ranolazine receiving group (*P* = .007) (Table 1). Of 90 patients receiving ranolazine, 54 (60.0%) were taking 1000 mg, 34 (37.8%) were taking 750 mg, and two (2.2%) were taking 1500 mg of ranolazine. Left ventricular end diastolic, end systolic, and left atrial diameters were higher in ranolazine group, whereas left ventricular posterior wall diameter was smaller (*P* < .001). However, left ventricular ejection fraction, left ventricular mass index, left atrial volume, and left atrial volume index were similar in the groups. White blood cell count, platelet count, total cholesterol, and AST values were importantly higher among laboratory values in the ranolazine group (*P* < .05) (Table 2). Acetyl salicylic acid and statin intake were higher in the control group, whereas nitrate and trimetazidine intake were significantly higher in the ranolazine group (*P* < .05). There was no significant difference between the groups in terms of calcium channel blocker, beta blocker, and other medical treatments (*P* > .05) (Table 2).

**TABLE 2:** Basic echocardiography, laboratory parameters and distribution of drug therapies among the study groups

Variable	Ranolazine (+) CAD group	Ranolazine (−) CAD group	*P* value
	(n = 90)	(n = 85)	
Ejection fraction (%)	50.6 ± 5.2	52.2 ± 6.1	0.136
LVEDD (mm)	50 (42‐64)	47 (37‐64)	**<0.001**
LVESD (mm)	33 (21‐42)	30 (18‐40)	**<0.001**
IVS (mm)	12.0 ± 1.5	12.5 ± 2.4	0.225
PW (mm)	10.1 ± 2.4	11.4 ± 1.5	**<0.001**
LV mass index (g/m^2^)	112.3 ± 26.1	109.8 ± 27.5	0.382
LA (mm)	42.7 ± 4.5	40.1 ± 4.1	**<0.001**
LAV (ml)	46.2 ± 13.7	44.2 ± 14.2	0.095
LA volume index (ml/m^2^)	28.0 ± 7.9	26.9 ± 8.4	0.082
Glucose (mg/dl)	118.5 (60‐350)	115.5 (74‐342)	0.401
Creatinine (mg/dl)	0.95 (0.6‐1.9)	0.88 (0.5‐2.0)	0.167
Haemoglobin (g/dl)	13.0 ± 1.4	13.5 ± 1.4	0.060
White blood cell (10^3^/ml)	8.5 ± 2.3	7.4 ± 1.7	**0.001**
Platelete (10^3^/ml)	258.5 (140‐650)	235 (128‐504)	**0.003**
Total cholesterol (mg/dl)	204.5 (105‐354)	175.4 ± 40.3 (100‐258)	**<0.001**
LDL (mg/dl)	104.5 (30‐252)	96.5 (24‐176)	0.145
HDL (mg/dl)	43.1 ± 10.1	43.6 ± 9.3	0.612
Triglyceride (mg/dl)	152.5 (70‐693)	150 (65‐526)	0.859
AST (Ul/L)	25 (11‐74)	20 (11‐77)	**0.001**
ALT (Ul/L)	23.5 (5‐72)	21 (7‐55)	0.085
Drugs, n (%)			
Beta‐blocker	73 (81.1)	74 (88.1)	0.204
ACEI/ARB	66 (73.3)	62 (73.8)	0.943
Acetil salicylic acid	56 (62.2)	70 (83.3)	**0.002**
Clopidogrel	58 (64.4)	51 (60.7)	0.611
Statin	69 (76.7)	78 (92.9)	**0.003**
Nitrate	24 (26.7)	9 (10.7)	**0.007**
Trimetazidine	31 (34.4)	16 (19.0)	**0.022**
Verapamil/diltiazem	3 (3.3)	2 (2.4)	1.000
Amiodarone	0	1 (1.2)	0.483
Digoxin	0	0	—
Ca‐channel blocker	23 (25.6)	17 (20.2)	0.405
Anti‐diabetic drugs	26 (28.9)	18 (21.7)	0.277
Ranolazine 750 mg	34 (37.8)	‐	
Ranolazine 1000 mg	54 (60)	‐	
Ranolazine 1500 mg	2 (2.2)	‐	

Note

Continuous variables are presented as “mean ± SD and median (minimum‐maximum)” and categorical variables as “number (percentage).”

Abbreviations: ACEI/ARB, angiotensin converting enzyme inhibitör/angiotensin receptor blocker; ALT, alanine transaminase; AST, asparte transaminase; EF, ejection fraction; HDL, high‐density lipoprotein; Hg, hemoglobin; IVS, interventricular septum; LA, left atrium‐parasternal long‐axis diameter; LDL, low‐density lipoprotein; LVEDD, left ventricular end‐diastolic diameter; LVESD, left ventricular end‐systolic diameter; PW, posterior wall; RV, right ventricle.

Bold values indicate statistical significance.

**FIGURE 1:**
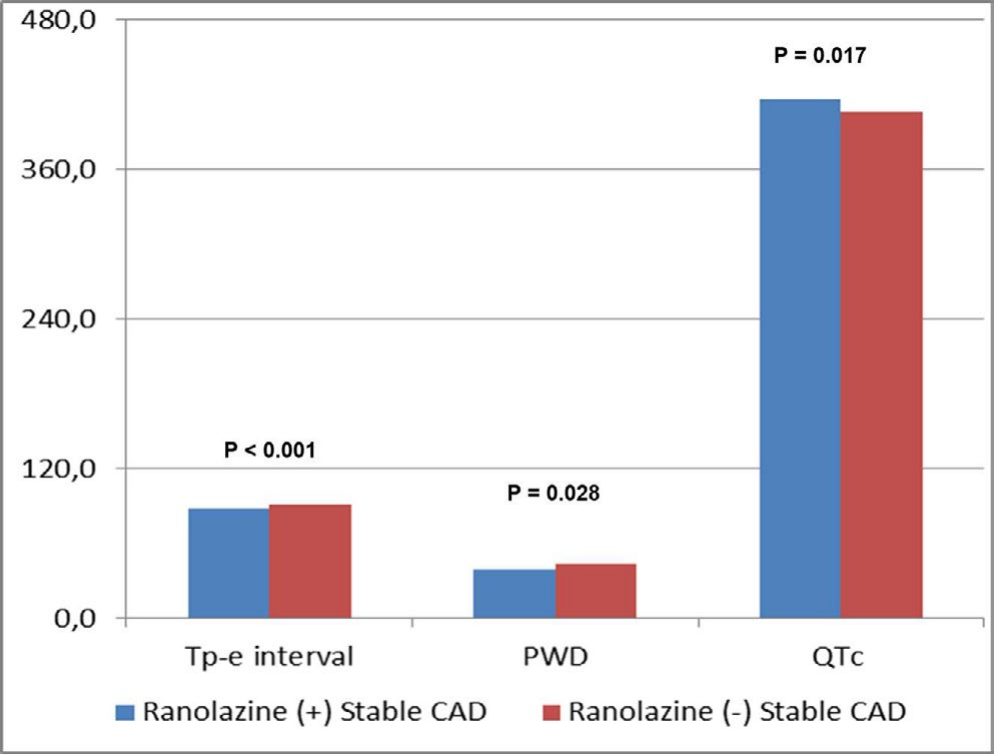
Imaging of Tp‐e interval, PWD, and QTc time between the groups

QTc time was significantly higher in ranolazine group (*P* = .017), but Tp‐e interval, Tp‐e/QT and Tp‐e/QTc ratios were significantly lower in ranolazine group than the control group (respectively, *P* < .001, *P* = .030, *P* = .001). PWD was also significantly lower in ranolazine group (*P* = .028) (Figure 1) (Table 3). In the multivariate logistic regression analysis, QTc, Tp‐e/QTc ratio, P_max_, and PWD were significantly difference in the groups again, after adjusting age, BMI, and heart rate parameters (respectively, *P* = .042, *P* = .021, *P* = .043, *P* = .037) (Table 4). There was no significant difference for iCEB (QT/QRS and QTc/QRS) between the groups (*P* > .05) (Table 3). In ranolazine group, when compared the myocardial repolarization parameters, PWD and iCEB, there was no differences between the patients using 750‐ and 1000‐mg doses of ranolazine (Table 5).

**TABLE 3:** Basic electrocardiographic parameters, repolarization parameters and P‐wave dispersion of the patients

Variable	Ranolazine (+) CAD group	Ranolazine (−) CAD group	*P* value
	(n = 90)	(n = 85)	
Heart rate (bpm/min)	71.5 ± 11.0	68.1 ± 9.7	**0.019**
PR (msn)	174.4 ± 24.6	171.5 ± 24.4	0.367
QRS (msn)	94.3 ± 10.9	95.5 ± 11.7	0.469
QT (msn)	392.4 ± 27.7	392.5 ± 26.5	0.804
QTc (msn)	417.5 (330‐465)	410 (345‐490)	**0.017**
Tp‐e interval (msn)	87.3 ± 14.4	90.8 ± 12.4	**<0.001**
Tp‐e/QT ratio	0.22 ± 0.04	0.23 ± 0.03	**0.030**
Tp‐e/QTc ratio	0.20 (0.14‐0.29)	0.21 (0.19‐0.32)	**0.001**
P_max_ (msn)	110.4 ± 15.9	115.4 ± 14.8	0.313
P_min_ (msn)	70.3 ± 10.8	73.8 ± 12.5	0.088
PWD (msn)	39.2 ± 13.7 (19‐74)	43.5 ± 12.9 (22‐77)	**0.028**
QT/QRS ratio	4.2 (3.27‐5.73)	4.1 (3.27‐5.43)	0.70
QTc/QRS ratio	4.40 (3.30‐5.73)	4.28 (3.47‐5.93)	0.09

Note

Continuous variables are presented as “mean ± SD and median (minimum‐maximum)” and categorical variables as “number (percentage).”

Abbreviations: P_max_, Pmaximum time; P_min_, Pminimum time; PWD, P‐wave dispersion; Tp‐e interval, Tpik to end interval.

Bold values indicate statistical significance.

**TABLE 4:** Logistic regression analysis to determine whether the effect of ranolazine is significant after adjusting age, BMI, and heart rate parameters

Variable	OR	95% CI	*P* value
PR (msn)	1.00	0.99‐1.02	0.578
QRS (msn)	0.99	0.96‐1.02	0.522
QT (msn)	1.00	0.99‐1.01	0.796
QTc (msn)	1.02	1.01‐1.03	**0.042**
Tp‐e interval (msn)	0.98	0.96‐1.01	0.118
Tp‐e/QT ratio	0.001	0‐12.23	0.148
Tp‐e/QTc ratio	0.001	0‐0.16	**0.021**
P_max_ (msn)	0.98	0.96‐0.99	**0.043**
P_min_ (msn)	0.97	0.95‐1.01	0.055
PWD (msn)	0.98	0.95‐0.99	**0.037**
QT/QRS ratio	0.04	0‐899.9	0.535
QTc/QRS ratio	0.01	0‐17.88	0.142

Abbreviations: CI, confidence interval; OR, odds ratio.

Bold values indicate statistical significance.

**TABLE 5:** Evaluation of myocardial repolarization parameters, PWD and iCEB in patients using 750‐ and 1000‐mg doses of ranolazine in the ranolazine group

Variable	Ranolazine 750 mg (+) CAD group	Ranolazine 1000 mg (+) CAD group	*P* value
	(n = 34)	(n = 54)	
Heart rate (bpm/min)	70.1 ± 10.6	72.7 ± 11.0	0.295
PR (msn)	173.7 ± 24.6	175.9 ± 24.1	0.700
QRS (msn)	96.7 ± 11.7	93.3 ± 10.0	0.150
QT (msn)	395.1 ± 29.0	389.3 ± 27.2	0.344
QTc (msn)	417.6 (380‐460)	414.2 (330‐465)	0.511
Tp‐e interval (msn)	87.6 ± 13.6	88.9 ± 15.4	0.695
Tp‐e/QT ratio	0.22 ± 0.04	0.22 ± 0.03	0.524
Tp‐e/QTc ratio	0.21 (0.15‐0.28)	0.20 (0.14‐0.29)	0.539
P_max_ (msn)	109.1 ± 16.4	110.9 ± 16.1	0.619
P_min_ (msn)	67.6 ± 10.6	72.1 ± 10.8	0.061
PWD (msn)	41.4 ± 13.3 (19‐74)	38.7 ± 14.2 (19‐74)	0.379
QT/QRS ratio	4.1 (3.27‐5.35)	4.2 (3.27‐5.73)	0.469
QTc/QRS ratio	4.37 (3.30‐5.73)	4.49 (3.30‐5.73)	0.292

## 4 DISCUSSION

In this study, we evaluated the effect of ranolazine on repolarization parameters (Tp‐e interval, Tp‐e/QT ratio, QT, QTc), iCEB (QT/QRS and QTc/QRS), and PWD in patients with stable CAD. We found significantly lower Tp‐e interval, Tp‐e/QT ratio, Tp‐e/QTc ratio, and PWD in ranolazine therapy group compared with control group, but iCEB was similar in two groups. In multivariate logistic regression analysis, QTc, Tp‐e/QTc ratio, Pmax, and PWD were significantly difference between the groups again, after adjusting confounding parameters.

Ranolazine inhibits late sodium (Na) inflow channels and prevents indirect intracellular calcium (Ca) accumulation. This effect reduces ventricular diastolic tension and oxygen consumption and improves symptoms in stable angina pectoris patients. Ranolazine also causes partial inhibition of fatty acid oxidation.^3^, ^11^ In addition to Na channels, ranolazine also inhibits late rectifying potassium flow, and some other currents led to cause in the antiarrhythmic effect. Ranolazine causes a minimal prolongation in the action potential duration as a result of its effect on the potassium flow. Minimal extension in QTc is independent from heart rate. Triggered activity and torsades de pointes type ventricular tachyarrhythmia's are not expected due to the presence of early post‐depolarization and transmural repolarization dispersion.^11^, ^12^, ^13^, ^14^ Antzelevitch et al.^15^ showed that ranolazine caused a dose‐dependent delay of the action potential in epicardial area but shortened the action potential in mid‐myocardial area, so caused to decline or no change in the total transmural distribution of repolarization. Again, Evaristo et al.^16^ showed that ranolazine reduced repolarization heterogeneity in symptomatic patients with diabetes and nonflow‐limiting coronary artery stenosis. However, ranolazine is contraindicated in patients who have QT prolongation or taken drugs which cause QT prolongation.^11^ In our study, QTc was found significantly prolongation in patients receiving ranolazine (416.0 ± 22.3 vs. 408.5 ± 20.8 msn; *P* = .017) without effect antiarrhythmic agents between groups.

Although ranolazine is not among the direct antiarrhythmic agents, the MERLIN‐TIMI 36 study showed that the drug may have antiarrhythmic effects. In this study, it was shown that the incidence of VT, supraventricular tachycardia and AF decreased by 3%, 10.3%, and 0.7%, respectively.^17^. In the RAID study, ranolazine did not decrease the incidence of the first VT, VF, or death in high‐risk ICD patients, but it was related with an important decrease in repetitive VT or VF in patient who was requiring ICD therapy ^18^. In our study, the data of stable CAD patients were cross sectional. Therefore, long‐term effects of ranolazine could not be obtained our data.

Murdock et al ^3^, ^19^ recently demonstrated in a case series that ranolazine may be helpful in the treatment of electrical cardioversion in patients with cardioversion‐resistant AF. In another study showed that adding of ranolazine to amiodarone was safe, well tolerated, and effective, superior to only amiodarone for conversion of new‐starting AF.^20^ An increase in PWD shows irregular intra atrial and interatrial distribution of sinus beats and contributes a substrate for reentry mechanisms and new onset AF.^6^, ^21^, ^22^, ^23^ In our study, PWD was significantly lower in stable CAD patients with ranolazine therapy (39.2 ± 13.7 vs. 43.5 ± 12.9 msn; *P* = .028). Also, in previous studies, increased left atrial diameter has been associated with increase in PWD and PAF.^21^, ^24^ In our study, LA diameter was higher in receiving ranolazine group, but left atrial volume and left atrial volume index similar in groups. PWD was lower in the ranolazine group, regardless of left atrial diameter and volume, so these findings support the results of previous studies.

Lethal arrhythmic events were thought to be induced by electrophysiological disturbances of ventricular repolarization.^25^. QT and QTc intervals reflect both depolarization and repolarization. Prolongation of QT and QTc times has been used traditionally as a risk factor for lethal arrhythmic events. The T peak to end (Tp‐e) interval reflects total trans‐myocardial distribution of repolarization, so increased of Tp‐e interval is related and more predicted with malign ventricular arrhythmias and sudden cardiac death.^26^, ^27^, ^28^ Tp‐e/QT ratio is more sensitive index for arrhythmic events, because it is not affected by BMI, heart rate variability, or potential interpersonal changes of QT interval.^28^ Increased Tp‐e/QT ratio is related with arrhythmic events due to increased re‐entrant pathophysiological mechanism.^28^ In healthy population, Tp‐e/QT ratio calculated in the V5 and D2 leads, which best reflects the apicobasal and interventricular distribution of repolarization, has an average value of 0.21 ± 0.03 and an interval of 0.15‐0.25.^28^ Previous studies have shown that Tp‐e/QT ratio was significantly higher in many diseases, such as long and short QT syndrome, Brugada syndrome, early repolarization pattern, acute myocardial infarction, mitral valve prolapse, coronary slow flow, aortic stenosis, and extremely obesity.^7^, ^9^, ^11^, ^25^, ^26^, ^27^, ^28^, ^29^, ^30^, ^31^ In the literature, there was no study showing the relationship between the repolarization parameters and ranolazine therapy. In our study, Tp‐e interval, Tp‐e/QT, and Tp‐e/QTc ratios were significantly lower in stable CAD with ranolazine therapy (respectively, *P* < .001, *P* = .030, *P* = .001). Also, QTc and Tp‐e/QTc ratio were significantly different between the groups after excluding confounding factors (respectively, *P* = .042, *P* = .021).

Recently, iCEB was described as a new, non‐invasive feature, which may anticipate malign ventricular arrhythmias.^8^, ^31^ It is similar to cardiac wave‐long (λ = effective refractory period [ERP] × conduction velocity [CV]) and increased or decreased iCEB parameters are related with ventricular proarrhythmic risk.^8^, ^31^ In previous studies, iCEB was shown to change significantly in the opposite directions in patients with Brugada and Long QT syndrome, and both were detected to increase the risk of ventricular arrhythmias.^8^, ^31^ In our study, there was no important variation in the iCEB value among the groups (*P* > .05). Since iCEB is obtained with QT time divided by QRS time, it may not be significant in our study due to affecting both to ranolazine.

This study is important because the antiarrhythmic effects of ranolazine are supported by changes in ECG parameters in stable CAD patients. Our research also supports the outcomes of prior studies.

### 4.1 Study limitations

Our study had a small patient population. The main restriction of our study could not lack of long‐time follow‐up the patients and the inability to evaluate frequency of arrhythmic events between ranolazine (+) and control groups during follow‐up. We did not have the ECG before ranolazine treatment in ranolazine (+) group, so we could not analyze our study as before ranolazine, after ranolazine therapy, and control group. We had planned our study cross‐sectionally. Dynamic measurement of repolarization parameters better reflects the repolarization heterogeneity, but in our study, repolarization parameters were evaluated with the simplest, static, and easily accessible ECG. Also, in the ranolazine (+) group, CAD may be more complex and severe CAD, and this may be affecting the repolarization parameters.

## 5 CONCLUSION

We showed that ranolazine caused asymptomatic cardiac ECG changes in stable CAD patients. Tp‐e/QTc ratio, QTc, P_max_, and PWD were significantly lower in stable CAD patients under ranolazine therapy. In stable CAD patients, the prognostic significance of ranolazine for arrhythmic events requires further evaluation of these parameters through long‐term follow‐up and big‐scale prospective studies.

## 6 STATEMENT OF ETHICS

This study was approved by the Institutional Ethics Committee (Ondokuz Mayis University, ethic no: 2020/119). Informed, written consent was obtained from all patients. The study was performed in accordance with the Declaration of Helsinki.

## DISCLOSURE STATEMENT

The authors declare that they have no potential conflicts of interest concerning the research, authorship, and publication of this article.

## AUTHOR CONTRIBUTIONS

All authors contributed to the conception of the work and drafted the manuscript. All authors critically revised the manuscript and gave final approval.
